# Studies of the Mechanism of Nucleosome Dynamics: A Review on Multifactorial Regulation from Computational and Experimental Cases

**DOI:** 10.3390/polym15071763

**Published:** 2023-04-01

**Authors:** Danfeng Shi, Yuxin Huang, Chen Bai

**Affiliations:** 1 Warshel Institute for Computational Biology, School of Life and Health Sciences, School of Medicine, The Chinese University of Hong Kong (Shenzhen), Shenzhen 518172, China; shidanfeng@cuhk.edu.cn (D.S.); 120030024@link.cuhk.edu.cn (Y.H.); 2 School of Chemistry and Materials Science, University of Science and Technology of China, Hefei 230026, China; 3 Chenzhu (MoMeD) Biotechnology Co., Ltd., Hangzhou 310005, China

**Keywords:** nucleosome dynamics, mechanism studies, multifactorial regulation

## Abstract

The nucleosome, which organizes the long coil of genomic DNA in a highly condensed, polymeric way, is thought to be the basic unit of chromosomal structure. As the most important protein–DNA complex, its structural and dynamic features have been successively revealed in recent years. However, its regulatory mechanism, which is modulated by multiple factors, still requires systemic discussion. This study summarizes the regulatory factors of the nucleosome’s dynamic features from the perspective of histone modification, DNA methylation, and the nucleosome-interacting factors (transcription factors and nucleosome-remodeling proteins and cations) and focuses on the research exploring the molecular mechanism through both computational and experimental approaches. The regulatory factors that affect the dynamic features of nucleosomes are also discussed in detail, such as unwrapping, wrapping, sliding, and stacking. Due to the complexity of the high-order topological structures of nucleosomes and the comprehensive effects of regulatory factors, the research on the functional modulation mechanism of nucleosomes has encountered great challenges. The integration of computational and experimental approaches, the construction of physical modes for nucleosomes, and the application of deep learning techniques will provide promising opportunities for further exploration.

## 1. Introduction

The nucleosome is the fundamental unit of the chromatin polymer, and each nucleosome contains a core particle, a short segment of linker DNA, and an associated linker histone. As the main binding block, the nucleosome core particle consists of a heterologous histone octamer and a stretch of wrapping DNA coil with ~146 bp [1]. The histone octamer is assembled by two replicas of four histones, including H2A, H2B, H3, and H4, which form two pairs of H3/H4 and H2A/H2B histone-fold heterodimers [2,3,4]. All four heterodimers are located in a relatively fixed positional relationship such that the H2A/H2B and H3/H4 dimers are arranged along a clockwise path as H3/H4 → H2A/H2B → H2B/H2A → H4/H3 to interact with the nucleosomal DNA from the disc view of the nucleosome [5]. The linker DNA is defined as the non-nucleosomal DNA connecting consecutive core particles with a length ranging between ~20 and 90 bp. The linker histone, with subtypes from H1.0 to H1.5 in the histone H1 family, directly interacts with the nucleosome core particle and linker DNA at the location around the nucleosomal DNA entry and exit sites [6].

**Figure 1:**
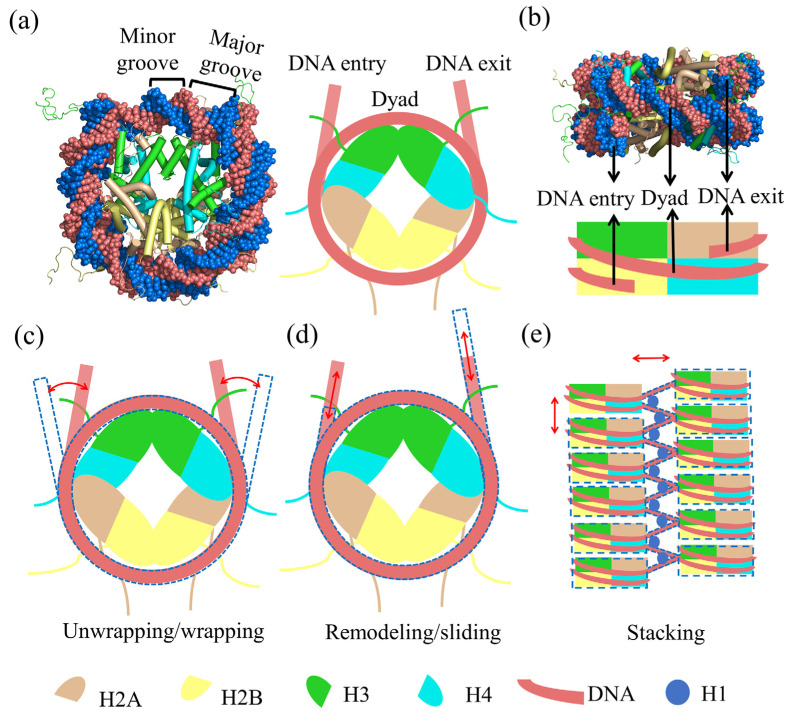
The schematic diagram for the structural basis and dynamic features of nucleosomes. (**a**) The disc-view schematic diagram of the nucleosome structures (PDB number: 1KX5) with histone H2A (brown), H2B (yellow), H3 (green), H4 (cyan), and the nucleosomal DNA (pink and light blue). The schematic diagram is shown next to the crystal structure to depict the topological relationship of the different components shown in the colored blocks. (**b**) The dyad-view schematic diagrams of the nucleosome structures. (**c**) The wrapping/unwrapping mode for the nucleosomal DNA. (**d**) The remodeling/sliding mode for the nucleosomal DNA. (**e**) The stacking mode for packing the nucleosome core particle, linker DNA, and linker histone (H1) into a dense state of nucleosomes.

The nucleosomal DNA coil wraps 1.65 times around the histone octamer with a pseudo-2-fold dyad axis passing through a single base pair at the center. It generates two adjacent DNA superhelical gyres on which major and minor grooves are aligned along the surface of the histone octamer. The superhelical locations (SHLs) are defined as positions at which the major groove of the DNA faces toward the histone surface. The SHLs are numbered, beginning with the nucleosomal dyad axis position (SHL0) with ~10 base-pair periodicity and ranging from SHL−7 to SHL+7. The nucleosomal DNA forms strong electrostatic or hydrogen-bonding interactions with residues on the histones mainly by its minor grooves [7]. Due to the heterogeneity of the histone–DNA interface, 14 separate interactive locations on the nucleosomal DNA have been detected, with stronger interactions at the dyad region and weaker interactions near the entry or exit site of the DNA [8]. All the N-terminal tails of the eight core histones protrude toward the bulk solvent and adopt flexible conformations [9]. As shown in Figure 1a,b, all these structural components together complete the nucleosome and lay a critical basis for the biological functions of the nucleosome. Nucleosomes can further interact with each other and form an ordered local chromatin architecture. As observed in crystal packing, nucleosomes tend to stack face-to-face with next-neighbor nucleosomes (i.e., between the nth and nth+2 nucleosomes) and form a tetranucleosome motif, which may function as a fundamental chromatin organizational unit. However, the chromatin structure is not uniform for contact between nucleosomes of various spacings, including (nth and nth+1), (nth and nth+2), and beyond, which may exist simultaneously due to the differences in linker DNA lengths. The 30 nm chromatin fiber is the higher-order level of the self-assembly of nucleosomes, which is thought to be the unit of higher-order packing for chromatin and is still being extensively researched [10,11].

As a multifunctional and comprehensive molecular machine, nucleosomes firstly provide the first level of DNA compaction; secondly, they act as a signaling hub for chromatin-templated processes such as transcription and DNA replication, repair, and recombination; thirdly, they support the self-assembly of higher-order chromatin structures [12]. According to diverse biological research studies and related review reports [13,14,15], these biological functions are mainly regulated by the dynamic conformational change of nucleosomes. As most interactions inside the nucleosome are relatively weak and occur mostly in the forms of electrostatic, hydrogen-bonding, and hydrophobic effects, their thermal energy is reported on the scale of ~0.6 kcal/mol [13]. Hence, nucleosomes could easily explore a broad conformational space related to their biological functions when facing the interference of regulatory factors. Currently, the dynamic features of nucleosomes have been revealed and presented as a variety of specific dynamic modes with obvious behavioral characteristics, as shown in Figure 1c–e. (1) Unwrapping/wrapping mode: the interaction between DNA and histones is weakened/strengthened and DNA is detached/bound with the histones, resulting in the exposure/burial of previously blocked DNA sites [16]; (2) remodeling/sliding mode: the change in the relative position between histones and DNA, accompanied by an interfacial interaction alteration [17,18]; (3) stacking mode: the packing of DNA and histones into a dense state of nucleosomes with spontaneous agglomeration and self-assembly, eventually forming a stable fiber structure [19,20].

These dynamic modes of nucleosomes have been demonstrated by a series of experimental approaches, including cryogenic electron microscopy (Cryo-EM), fluorescence resonance energy transfer (FRET), nuclear magnetic resonance (NMR) spectroscopy, and the force spectrometer. Cryo-EM is a microscopy technique in which samples are cooled to cryogenic temperatures (i.e., below −150 °C) for the determination of the 3D structures of biomolecules at near-atomic resolution [21]. FRET has become a highly sensitive reporter for intermolecular/intramolecular distances in living cells due to its compatibility in scale with biological molecules and the development of novel fluorophores and optical detection techniques [22,23,24]. NMR can also be applied for the determination of 3D structures, but it is more frequently applied to analyze protein interactions and dynamics at atomic resolution [25]. The DNA origami-based force spectrometer by Funke et al. enabled the measurement of nucleosome–nucleosome distance frequencies at sub-nanometer resolution with the use of imaging techniques [26]. Nevertheless, these experimental approaches could not provide detailed dynamic information at atomistic resolution, which is quite important for understanding the regulatory mechanism of nucleosomes in different biological processes. In light of the deficiency of the experimental approaches above, computational modeling and simulation can be applied as an alternative tool to complement experimental approaches, providing extra conformational characterization associated with the atomic interactions and dynamic features of nucleosome motion. A series of multi-scale simulations with different accuracies have been applied, including all-atom molecular dynamics (MD) at single-atom resolution, coarse-grained (CG) modeling at atom-group resolution, and mesoscale modeling at protein-particle resolution. Due to the large spatial and temporal scales of nucleosomes, the computational approaches have become important auxiliary means for mechanistic research and experiments [27,28,29,30].

Chromatin changes have significant effects on gene expression, DNA replication, and DNA repair processes which, in turn, can affect cell development, differentiation, and disease. For example, during the cell differentiation process, stem cells undergo changes in chromatin structure that allow them to adopt specific cell fates and functions [31]. Similarly, changes in chromatin structure have been implicated in a variety of diseases, such as cancer, in which abnormal chromatin modifications can lead to dysregulated gene expression and uncontrolled cellular replication [32,33,34]. The dynamic modes of nucleosomes determine the tight or loose state of the DNA wrapping on histones, which also reflects whether the chromosomes are currently in a closed state or an open state on the macro scale [35]. Therefore, revealing the regulatory mechanism of nucleosome dynamics may help to understand the disease and therapy. Current studies have shown that the regulatory factors of nucleosomes include histone modification, DNA methylation [31,36,37], and the intervention of nucleosome-interacting factors [38,39], as shown in Table 1. The dynamic modes of nucleosomes and the factors that affect these modes have already been reviewed from different aspects [40,41], and it has been suggested that the dynamic features of nucleosomes are often regulated by these factors, which are jointly and closely involved in genome regulation. This review is aimed at summarizing the solo or combination impact of different factors, and it describes the regulatory mechanism of nucleosome dynamics in a unified framework of three main factors. In this study, we mainly summarize the mechanism of the multifactorial regulation of nucleosome dynamics and provide an updated perspective using computational and experimental approaches. Through the comprehensive consideration of multiple regulatory factors, the constitutional, dynamic features of nucleosomes under physiological conditions can be revealed. The integration of different techniques and tools is also discussed for future research on nucleosomes.

## 2. Multiple Regulatory Factors and Associated Mechanism

### 2.1. Histone Modification

#### 2.1.1. Dynamic Features of Unmodified Histone

The dynamic features of histones are mainly reflected by the conformational fluctuations of the N-terminal tails of histones in their spatial arrangement [42]. These N-terminal tails, which range from 15 to 36 amino acids, are often exposed to the solvents outside the surface of their resident histones (in the case of H4 and H2A) or stretch through the channel between the superhelical gyres of the DNA (in the case of H3 and H2B). A dynamic change in conformation can extend the tails to a distance far from the core octamer or can form interactions with the DNA and histone protein at the intranucleosomal or adjacent nucleosomes [43]. Ohtomo et al. applied NMR to illuminate different conformations of the H2A and H2B tails of 145-bp and 193-bp nucleosomes, suggesting that the H2A N-terminal tail had stable locations at the major or minor grooves of nucleosomal DNA, while the H2B N-terminal tail had two different orientations toward or opposite to the entry/exit site. Further MD simulation indicated that the H2A N-terminal tail might have a stronger contact with the minor groove than the major groove, and the H2B N-terminal tail declined such contact in the major groove in both orientations toward and opposite to the entry/exit site [44]. To understand the structure and dynamics of the histone H3 N-terminal tail, FRET experiments were conducted to detect the motions of the H3 N-terminal tail versus the dyad axis and linker DNA. The result showed that H3 N-terminal tail interacted with DNA with certain dynamic transitions, which were accelerated by the charge-modifying mutations (R81E/R88E) on helix α3 of histone H2A. It was suggested that the multiple interaction modes of the H3 N-terminal tail could be regulated by the allosteric effects of the mutations within a distance less than 2–3 nm [24]. Rabdano et al. studied the interaction between the nucleosomal DNA and histone H4 tails in reconstituted nucleosomes based on the residue-specific ^15^N NMR rates. According to the simulated and experimental evidence, the NMR observables were reproduced in a 2-μs MD trajectory of the nucleosome. The H4 N-terminal tails tended to have highly disordered dynamics in spite of their reduced conformational flexibility, and they interacted with DNA in a complex and unclear way, supported by variable, short-lived salt bridges and hydrogen bonds at a low ionic strength [45]. Differing from the negative electrostatic characteristics of the center part of the histone octamer (also known as histone folds) [46], histone tails contain many arginine and lysine residues, demonstrating a strong and pure positive charge [47]. The highly charged density and low hydrophobicity of histone tails correspond to the characteristics of intrinsically disordered proteins (IDPs), which lack well-defined 3D structures and exist in a flexible, dynamic, and often disordered state [48].

The precise contributions of each histone tail to nucleosomes were evaluated by Iwasaki et al., who demonstrated that the deletion of the H2B or H3 N-terminal tail could affect histone–DNA interactions and decrease nucleosome stability [42]. High-amplitude breathing motions (wrapping/unwrapping) of the Lin28b and Esrrb nucleosomes (two genomic nucleosomes bearing the Esrrb and Lin28b enhancer sequences) and low-amplitude breathing motions of the engineered Widom nucleosome (a recombinant nucleosome bearing Widom 601 sequences) were further monitored by Huertas et al., using extensive sampling conformations by atomistic molecular simulations [49]. It was revealed that the absence of histone tails enhanced nucleosome breathing. As only the H3 N-terminal and H2A C-terminal tails were located near the last 15 base pairs of the DNA entry and exit sites, nucleosome opening and closing were proposed to be a direct result of the cooperative motions of these two tails. The molecular trajectories by Lequieu et al. also showed that the H4 tail was strongly co-localized with the DNA loops at θ ≈ ±π/2, stabilizing the DNA loops [50]. Hamiche et al. further proved the influence of histone tails on nucleosome sliding by reconstituting hybrid nucleosomes which lacked one or more histone N-terminal tails. The removal of the H2B N-terminal tail was unexpectedly found to promote uncatalyzed nucleosome sliding, and this effect could be enhanced by the additional removal of other histone tails [51]. A recent report by Lorch et al., which focused on the great abundance of positive charges in the histone tails, revealed that histone tails did not stabilize the core histone folds but rather were involved in the removal of the histone octamer, which has an essential function in chromatin remodeling [52]. It can be inferred that histone tails may impact the dynamic modes of nucleosomes mainly through their regulation of the interactions among histone monomers, between a histone octamer and nucleosomal DNA, and within adjacent nucleosomes. Due to the differences in structure and location among different histone subtypes, each histone subtype may display a specific dynamic effect of its histone tail; hence, the interplay between histone tails together can change the intra-nucleosome or inter-nucleosome interactions directly.

#### 2.1.2. Post-Translational Modifications of Histones

Post-translational modifications (PTMs) refer to the reversible or irreversible chemical modifications, primarily on the amino acid side chains of a polypeptide chain, which can change original localizations or activities. On histones, PTMs are mainly located in four different domains: histone tails, the dyad symmetry axis region, the DNA entry/exit region, and interfaces between histone dimers [53]. The modifications added to the histone residues can be as small as chemical groups, such as methyl (on lysine, arginine, and glutamine), acetyl (on lysine) and phosphoryl groups (on serine and threonine), or as large as the entire ubiquitination protein. Among different types of PTMs, lysine acetylation, serine/threonine phosphorylation, and lysine ubiquitination can directly change the structure of chromatin, while lysine and arginine methylation often act as a signal hub, targeted by the proteins in the nucleus. Both acetylation and phosphorylation reduce the positive charges of histone, thereby weakening the electrostatic interaction with negatively charged DNA. The acetylation of histone tails is also involved in the disruption of nucleosomal arrays in chromatin, and the modified tails of the unfolded array can further interact with other macromolecules during the transcription process [54]. Ikebe et al. conducted enhanced sampling simulations to investigate the effect of acetylation on the conformations of H3 histone tails [55]. The result indicated that acetylation of histone H3 lysine 14 (H3K14) lightly reduced the interaction between the H3 tail and the nucleosomal DNA and enabled the H3 tail to form a more compact α-helix structure, which resulted in the exposure of linker DNA and facilitated the binding of transcription factors and other DNA-binding proteins to their target sequences. The ubiquitination of histone can relieve the tight stacking of the nucleosome, which contributes to transcription and DNA damage repair [56]. It has been indicated that all the above-mentioned PTMs on histones can directly change the biophysical characteristics of histones and induce the conformational change of DNA gyres, which provide an accessible site for other proteins [57,58].

#### 2.1.3. The Interplay of Different Histone Modifications

The interplay between histone tails, accompanied by modifications, can affect the dynamics of nucleosomes. The probability of altered conformational states of the nucleosome could be highly enriched by PTMs [59,60]. Forties et al. compared the influence of different sites and types of histone PTMs near the nucleosome dyad, including the phosphorylation of histone H3 threonine 118 (H3T118) and the double acetylation of histone H3 lysine 115 (H3K115) and histone H3 lysine 122 (H3K122), suggesting that the dyad modifications could increase the probability of an unwrapping fluctuation that allows for the release of a histone octamer [61]. A series of studies also demonstrated that the post-translational modifications of histone tails could significantly change the inter-nucleosomal interactions and thus alter the nucleosome stacking and condensation [58,62]. According to an in silico research study by Norouz et al., changing the stacking interaction on the scale of a few kcal/mol was sufficient to transform the chromatin from an open state into a compact fiber [63], which was also consistent with the tension-dependent free energy evaluation performed by Lequieu et al. [64]. Chen et al. applied the Bayesian network model to reveal inter-nucleosomal communication between histone modifications for nucleosome phasing. H2A variants and histone H4 lysine 20 mono-methylation (H4K20me1) on neighboring nucleosomes showed a novel specific epigenetic interaction, and their negative correlational relationship was strongly correlated with the size of the nucleosome-free region, and the strength of nucleosome phasing around transcription termination sites [65].

### 2.2. DNA Methylation

#### 2.2.1. DNA Methylation Effect on DNA Properties

DNA methylation is a heritable epigenetic mark and can also occur on nucleosomal DNA [66]. During early embryonic and germline development, DNA methylation is erased and re-established throughout the genome in a cell-type-specific manner. Once established, this de novo DNA methylation is maintained to preserve cellular identity [67,68]. This modification could affect the interaction between the transcription factor and DNA [69] or regulate gene expression by recruiting specific histone modifiers and chromatin remodelers [70]. Basically, the DNA skeleton in the periphery of the nucleosome shows a high degree of negative electricity. Most DNA methylations transfer the methyl group to cytosine nucleotides that precede a guanine nucleotide, known as CpG sites, through DNA methyltransferases (DNMTs) [71]. The addition of a methyl group to the C-5 position of a cytosine that points to the major groove of DNA will affect the geometric characteristics of the DNA by changing the hydrophobicity. Other DNA modifications, including 5-hydroxymethylcytosine (5hmC), 5-formylcytosine (5fC), and 5-carboxycytosine (5caC), are considered to be oxidized derivatives of 5mC under the catalysis of ten-eleven translocation (TET) proteins [72]. As an important intermediate for DNA demethylation, 5hmC could reshape the DNA methylation landscape and positively correlate with enhancer activities and chromatin accessibility, and its regulatory machinery has also been under vigorous investigation [73,74,75].

A series of molecular dynamics simulation research studies and experiments have studied the effect of methylation on the physical properties and dynamic features of DNA. Battistini et al. performed molecular dynamics simulations, biophysical experiments, and NMR spectroscopy to study the impact of 5-hydroxymethylation on DNA cytosine and demonstrated that the modified cytosines (5mC or 5hmC) made the DNA stiffer than the normal cytosine [76]. Further studies performed by Hognon et al. also explained how different levels of methylation in CpG sites affect the behavior of DNA [77]. Using full-atom molecular dynamics simulations and electronic circular dichroism for the unmethylated, hemi-methylated, and fully methylated *adenomatous polyposis coli* (APC) promoting region, it was concluded that the extended methylations of CpG sites could significantly alter the DNA backbone torsional parameters (especially the ζ angle, defined as C3′-O3′-P-O5′) in a cooperative way and change the accession to the major groove of DNA. A strong combinatorial effect of methylation and sequence context was also observed through the analysis of the additional energy cost from the underwinding or overwinding of DNA strands [78]. It was illustrated that the DNA response to torsional stress induced by methylation was heterogeneous due to the difference in sequence environment. The similar heterogeneous effect of methylation on the strand separation of DNA was also achieved by Severin et al. through the application of single-molecule force experiments and simulation [79]. More detailed conformational changes at atomic scale were systematically investigated by Carvalho et al. and Kameda et al. [80,81]. The double-stranded DNA system with different methylation sites and methylated CpG content was analyzed with respect to the characteristic changes in terms of local flexibility and the relative positioning of the nucleotides (base-step variables), showing the difference in the overall geometry and local flexibility at each base step, including shift, tilt, or twist movements.

#### 2.2.2. DNA Methylation Effect on the DNA–Histone Complex

DNA methylation can significantly affect the interaction between histones and nucleosomal DNA, and it also participates in the adjustment of the three-dimensional chromatin structure. Buitrago et al. inspected the intrinsic impact of DNA methylation by knocking in the DNA methylation machinery into a model system deprived of any cytosine methylation. They demonstrated that methylation impacted the structure of the chromatin both locally and globally [82]. The chromatin structure could also influence de novo DNA methylation. It has been suggested that the nucleosomal complex of histone and DNA are generally stable, and nucleosome occupancy is a major determinant of global DNA methylation patterns [83]. Experiments in vitro and in vivo suggested that nucleosomal DNA had a 2-fold decrease in CpG methylation compared to the linker regions of the nucleosomes [84]. It has been indicated that methylation may result in distinct effects on the dynamic features of nucleosomes due to different positions of methylated cytosines, various DNA sequence contexts, or experimental conditions [69]. Li et al. quantified the effects of DNA hypermethylation on nucleosomes in a time-domain fluorescence lifetime measurement. The DNA breathing motion was not more constrained with extensive CpG methylation than the normal DNA; however, the conformational equilibrium of nucleosomes was found to be more open due to a reduction in DNA backbone rigidity upon the addition of methyl groups [85]. The existence of methyl groups was deduced to account for the observed structural and dynamic differences of nucleosomes. Lee et al. compared the FRET efficiency of the Me601+39 nucleosome and the unmethylated counterpart Ume601+39, indicating that the tighter wrapping of DNA around the histone was induced by CpG methylation and accompanied by a topology change [86]. A molecular dynamics study by Portella et al. focused on the methylation of CpG steps in sites of the DNA minor groove that faced the histone core [87]. It was believed that methylated CpG steps demonstrated greater stiffness, which was the key factor for the decrease in the stability of the nucleosome. Li et al. applied microsecond-scale molecular dynamics to investigate the dynamics effects of cytosine methylation at CpG sites on nucleosomes [88]. A more curved, under-twisted DNA that narrowed the adjacent minor grooves was observed to shift the population equilibrium of the sugar phosphate backbone geometry. The methylation induced pronounced changes in geometry for both linker and nucleosomal DNA, including the change in the unwrapped base pairs and the twist, roll, and bending angle parameters. Its interactions with the histone octamer were also characterized by the contact and distance analyses between the histone and DNA, showing a higher stability and compactness in methylated systems than in unmethylated ones. It has been suggested that DNA methylation on nucleosomes could affect the physical properties of both linker and nucleosomal DNA, which, in turn, influence the propensity and the strength of the interactions with histones and further change the dynamic features of nucleosomes. Understanding how DNA methylation affects nucleosomes could provide new insights into how genes are repressed by inducing a nucleosomal closed and rigid state, thereby blocking the interaction between transcriptional activators or repressors and methylated CpGs [89].

### 2.3. Interactions of Nucleosome-Interacting Factors

#### 2.3.1. Transcription Factors

Transcription factors (TFs) act as adaptor proteins that identify specific DNA motifs and regulate the target genes [90]. As the gatekeeper of the TF binding site, nucleosomes can restrict the accession of transcription factors and control the inherent transcription process [91]. TFs are often bound to a partial DNA motif on nucleosomes with a general binding mode, which can be recognized as a short α-helix from the TFs anchoring on and protruding into the major groove on nucleosome DNA [92,93,94]. This binding mode is exemplified by a series of TFs such as FOX [95], ETS [96], zinc finger factors [97,98], and homologous domain transcription factors [92]. According to the binding strength between the DNA-binding domains of TF and nucleosomal DNA, TFs can be classified into strong nucleosome binders (group I) and weak nucleosome binders (group IIA and group IIB) [92]. It has been indicated that the interactions of TFs with nucleosomes can also be achieved by directly competing with nucleosome histones or recruiting active chromatin remodelers, which can evict nucleosomes dynamically [99]. Most TFs have more access to free DNA than nucleosomal DNA, and they share generally similar motifs bound to nucleosomes. According to a systematic research study on the interactions between the nucleosome and 220 TFs from diverse structural families, the binding positions of TFs on nucleosomal DNA are close to the end of the nucleosomal DNA or the periodic position of the solvent-exposed side [100]. When approaching the nucleosome gradually, TFs could destroy the stability of the near-end nucleosomal interactions, establish a stable interaction with nucleosomal DNA, and obtain access to genetic information on the DNA [101,102,103]. The increasing concentration of competitive TFs against histones around the local nucleosomes enhances the access to nucleosomal DNA for other TFs and auxiliary factors.

A unique class of TFs, known as pioneer TFs, can bind to DNA in closed chromatin contexts and help open closed chromatin to activate gene expression [104]. Oct4, Sox2, and Klf4 are three pioneer TFs known for their cooperation and ability in the conversion of somatic cells to pluripotent stem cells [105]. The complex of transcription factors Oct4 and Sox2 were discovered to preferentially bind at two different binding sites on nucleosomes [94]. An Oct4–Sox2 complex could remove DNA from histones (H2A/H3) when bound at SHL−6, while it only induced local DNA distortions when bound at SHL+6. Sox2 TFs alone were also reported to bind and locally distort DNA at SHL+2, which facilitated the detachment of terminal nucleosomal DNA [106]. It was suggested that the binding preference of pioneer TFs at different binding site positions could distort nucleosomal DNA differentially and provide access to different chromatinized motifs. Kim et al. applied transcription factors as perturbation probes to investigate nucleosome dynamics in living cells and built a stochastic model that accounted for nucleosome eviction by TF activity. The binding site of TF affected the eviction probability of the nucleosomes, and the effect on the eviction probability was preferentially observed when TFs were bound to locations adjacent to the symmetry axis [107]. Huertas et al. built a structure of Oct4 bound to Lin28b with two potential binding sites from experimental data and carried out microsecond-timescale MD simulations. The atomic resolution provided clues that the amplitude of nucleosome motions such as breathing and twisting were increased by the binding of Oct4 to multiple TF binding sites with a higher local structural flexibility [108]. A detailed regulatory mechanism of nucleosome dynamics by TF was proposed by MacCarthy et al., who suggested that Oct4 altered the optimal wrapping of the two gyres around each other and the histones but did not mediate nucleosome opening [109]. It has been indicated that intrinsic nucleosome flexibility is important for Oct4 binding, and the magnitude of Oct4’s impact on nucleosome dynamics is dependent on the binding site position and the mobility of histone tails.

#### 2.3.2. Nucleosome Remodeling Proteins

Nucleosome remodeling proteins, also known as ATP-dependent chromatin remodelers, can manipulate a series of dynamic movements of nucleosomes, including DNA sliding, ejection, or the incorporation of histone variants [110,111]. These remodelers have evolved and existed in all eukaryotic creatures, from yeast to human beings, and they can be classified into nucleosome translocation enzymes that slide DNA along histones and histone exchange factors that can physically exchange protein variants of histones or remove the entire histone core [17]. The nucleosome translocation enzymes are highly conservative and are usually divided into imitation switch (ISWI) [112], chromodomain helicase DNA-binding (CHD) [113], switch/sucrose non-fermentable (SWI/SNF) [114], and INO80 [115] subfamilies. By using the energy of ATP hydrolysis, these nucleosome remodelers are capable of reconfiguring DNA–histone interactions by either assembling, disrupting, or moving nucleosomes [110]. The dynamic mode of remodeling/sliding has a common characteristic of enzymatic reactions that is ATP-dependent and applied to break the histone–DNA contact in nucleosomes [116].

Chromatin remodelers could alter the position and composition of nucleosomes and were involved in a unifying a fundamental mechanism of DNA translocation, according to the investigations on a series of ATP-driven remodeling enzymes including Iswi, Chd1, and Ino80 [117]. Previously, a perturbation theory was applied to evaluate the effect of these enzymes through the construction of effective equilibrium models with rescaled temperatures and interactions, and it was proven to be accurate in predicting kinetic and steady-state quantities [118]. Brandani et al. applied means of molecular dynamics simulations to investigate the molecular mechanism of active nucleosome sliding in the system of the Snf2 remodeler complexed with a nucleosome. An inchworm mechanism was proposed in which DNA sliding began from the remodeler binding location and propagated to complete sliding throughout the entire nucleosome via the generation of a pair of twist defects [119]. Furthermore, this mechanism was investigated using coarse-grained molecular simulations and Markov state modeling in the sliding process of 601 nucleosomes by ∼10 bp. The typical times to observe sliding by 1 bp with uniform sequences were 0.013, 0.026, 0.09, 0.45, 0.27, and 0.8 s for the poly-AC, AT, AG, CG, CC, and AA sequences, respectively. It was indicated that the sliding dynamics were dependent on the sequence heterogeneity of nucleosomal DNA with different twist defect energies. It was also observed that nucleosome sliding was associated with not only nucleosomal defects that corresponded to a missing base pair at one SHL or commonly existed in crystal structures but also to defects characterized by DNA over-twisting with an extra base pair compared to canonical nucleosomes [120]. The structural rearrangements of the histone octamer could also affect the DNA sliding. Bilokapic et al. observed different conformational states of a histone octamer with distortion of the overall nucleosome, and they revealed that the strain distorting and moving the DNA at SHL+2 was induced by rearrangements in the histone core α-helices and DNA [121]. Bhardwaj et al. further demonstrated the effect of yeast Isw1a remodeler on chromatin at the higher-order structural specificity level, which was beyond the regulations at the level of single nucleosomes. When bound to dinucleosomes, Isw1a induced large allosteric changes that activated the nucleosome modeling and spacing activities, which were required for proper chromatin organization [122].

#### 2.3.3. Cations

Except for the surrounding proteins, ions are also important environmental factors that regulate the dynamics of nucleosomes. Magnesium is a necessary metal element in human beings and mainly takes part in biological processes in its ion form (Mg^2+^) [123]. As the most common metal co-factor in human cells, Mg^2+^ is involved in the hydrolysis of Mg^2+^-chelating ATP, and the regulation of the catalytic activity or structural stability of RNA, DNA, and protein enzymes [124,125]. Mg^2+^ was first found to affect DNA self-assembly in 2007, an effect which was independent of the conformational or mechanical properties [126]. More recently, it was reported that nucleosomes can sense the concentration of divalent cations such as Mg^2+^ and Ca^2+^ in their surroundings and preferentially associate with a similar phenomenon as nucleosome self-assembly [127,128]. With the reveal of the effect of Mg^2+^ on the higher-order structures of chromatin and chromosomes, Mg^2+^ turned out to be an important regulator for nucleosome dynamics and chromatin-based biological processes [129]. Except for the multivalent ions above, monovalent ions such as Na^+^/K^+^ were also reported to affect the characteristics of nucleosomes.

The nucleosome–nucleosome attraction could be regulated by the surrounding ion concentration. Sun et al. began to simulate the effect of multivalent cations for systems comprising 20 nucleosome core particles, and the results of the coarse-grained (CG) model simulation revealed that the participation of positively-charged histone tails and multivalent (Mg^2+^, Co^3+^) and monovalent (K^+^) ions was necessary for DNA–DNA and core–core interactions and enabled the close stacking of nucleosomes [130]. Andreeva et al. further compared the effects of Na^+^ and K^+^ ions on nucleosome structure, stability, and interactions with proteins. The stabilizing effect of K^+^ was noticeably higher than that of Na^+^ and was accompanied by a maximal stabilizing effect on nucleosomes at a concentration of 80–150 mM, while Na^+^ supported a more efficient reorganization of the nucleosome structure by poly(ADP-ribose) polymerase 1 and ATP-independent uncoiling by FACT when compared to K^+^ [131]. Another modeling study also demonstrated that ions could accumulate at the site between the nucleosomal DNA gyres, which would prevent nucleosome breathing [132]. Based on the net charge regulation of the ion atmosphere around nucleosomes, Gebala et al. provided a quantitative comparison approach to evaluate the net electrostatic fields by determining the number of ions associated with free double-stranded DNA and with nucleosomes. It was indicated that the net electrostatic field was still strong even if the net charge of the nucleosome was much less than that of the free DNA, and this high overall negative electrostatic field controlled the DNA compaction and chromatin function [133].

## 3. The Crosstalk of Multiple Factors and Effects on the High-Order Structure of Nucleosomes

**Figure 2:**
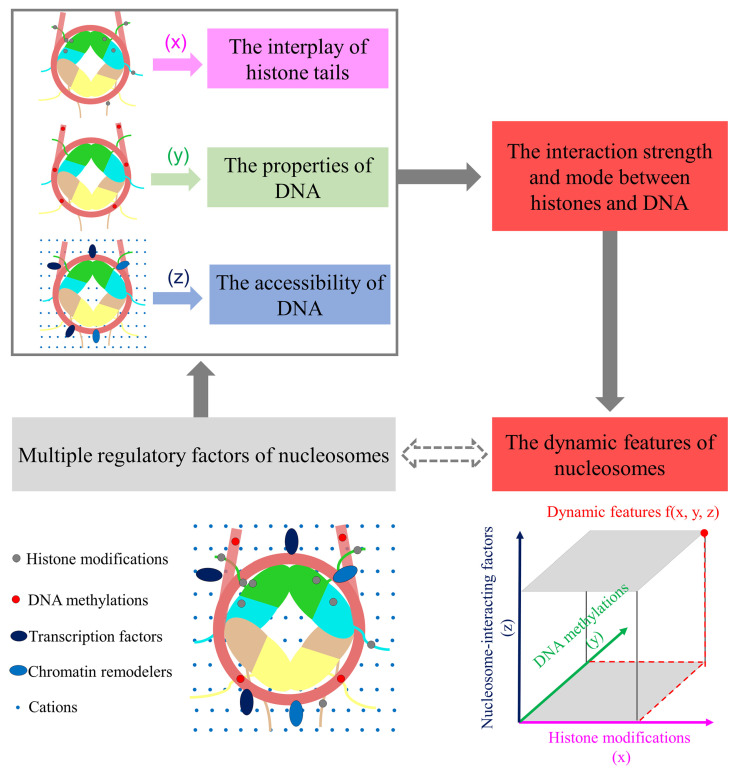
The main regulatory mechanism of nucleosome dynamics by the impact of different regulatory factors. The regulatory factors consist of histone modifications (x), DNA methylation (y), and nucleosome-interacting factors (z), including transcription factors, chromatin remodelers, and cations. Three regulatory factors are shown in colored blocks and distributed in different locations on nucleosomes. The dynamic features of nucleosomes are regulated comprehensively by the impact of histone modifications (x), DNA methylation (y), and nucleosome-interacting factors (z), and the effect of the dynamic features is depicted as f(x, y, z). The arrow icon in solid line represents the current strategies for the mechanism research of nucleosomes, while the arrow icon in dash line represents the possible strategies for the mechanism research of nucleosomes in future.

In real physiological circumstances, the dynamics of nucleosomes are simultaneously regulated by histone modifications, DNA methylation, and different interacting proteins or ions, as shown in Figure 2. Generally, the combination of these regulatory factors determines the dynamic characteristics of the nucleosome and further affects the diverse biological process. Bartke et al. began to identify “cross-talk” between histone modifications and DNA methylation in 2010. Stable isotope labeling by amino acids in a cell culture (SILAC) is a proteomic technique based on the incorporation of normal essential amino acids (light label) and isotopically modified amino acids (heavy label) into a cell culture. It can be used as a quantitative proteomic approach in any cell culture system. An affinity assay and a SILAC-based proteomic analysis were conducted to reveal the protein binding to nucleosomes regulated by the methylation of CpG sites, histone H3 lysine 4 (H3K4), histone H3 lysine 9 (H3K9), and histone H3 lysine 27 (H3K27), or a combination thereof. A cooperative, stronger binding of UHRF1 to histone H3 lysine 9 tri-methylation (H3K9me3)-modified nucleosomes was observed in the presence of CpG-methylation, while the incorporation of CpG-methylation into histone H3 lysine 27 tri-methylation (H3K27me3)-nucleosomes could counteract the recruitment of the PRC2 complex [134]. Brandani et al. also provided insights into the kinetics of nucleosome assembly when considering the influence of salt concentration and the heterogeneity of the DNA sequence. The results from Markov state model (MSM) models showed a clear asymmetry between the left and right side of the 601 sequence and the difference in nucleosome assembly pathways, suggesting the importance of the sequence-dependent shape and flexibility of DNA as well as the ion concentration [135]. A detailed summary of the impact of various factors on the conformational fluctuations of nucleosomes was also reviewed by Brandani et al., indicating the comprehensive regulation of nucleosomes by different factors is significant for nucleosomes [136].

Except for the direct effects on the chemical and physical properties of DNA and histones, PTMs and DNA methylation also change the recruitment of remodeling complexes in an indirect way and further affect the nucleosome dynamics together. The PTMs, including the methylation of histone H3 lysine 4 (H3K4), histone H3 lysine 9 (H3K9), histone H3 lysine 27 (H3K27), and histone H3 lysine 36 (H3K36), have a close connection with DNA methylation [137]. In the case of histone H3 lysine 4 (H3K4), the methylation effect of DNA methyltransferase 3 Like (DNMT3L) could be strongly inhibited by the methylation at histone H3 lysine 4 (H3K4), but it was insensitive to modifications at other positions [138]. Jie Li et al. systematically reviewed the methylation of histone H3 lysine 36 (H3K36) and its recruitment of different “reader” proteins and involvement in mediating chromatin remodeling or gene transcription regulation during various chromatin-templated contexts [139]. It was indicated that the molecular recognition between PTMs and chromatin-remodeling complex is required for nucleosome recruitment. Jian Li et al. further reported the binding preference of the Ioc4-PWWP domain of Isw1b chromatin-remodeling complex with histone H3 lysine 36 tri-methylation (H3K36me3) and revealed the structural basis for the recognition [140]. Cynthia Tallant et al. also reported the recognition preference of different domains of chromatin remodeling complex NoRC for different PTMs [141]. Currently, no conclusion has been reached as to whether PTMs, DNA methylation, and the effect of the recruited remodeling complexes are synergistic with or in opposition to the regulation of nucleosome dynamics [53].

The dynamic characteristics of single nucleosomes will further affect the formation of the high-order structures of nucleosome assembly. Zhao et al. compared the assembly of canonical and variant histone tetramers and octamers through a molecular dynamics simulation and concluded that variant histone CENP-A was thermodynamically favorable for a tetramer formation, whereas the canonical H3 at the tetramer interface presented remarkable swiveling dynamics which contributed to a rugged yet shallow binding free energy landscape [142]. Alvarado et al. further explored the manner of nucleosome packing at mesoscopic scales and observed two characterized tetranucleosomal conformations (“α-tetrahedron” and “β-rhombus”), suggesting that the local inter-nucleosomal interactions drove the formation of tetranucleosome motifs, which supported a mechanistic process of chromatin packing, dynamics, and accessibility that was strongly affected by the emergent local mesoscale structure [143]. By applying a transferable, enhanced-sampling Debye-length replica-exchange molecular dynamics approach, Farr et al. sampled chromatin at a high resolution. It was uncovered that nucleosome thermal fluctuations favored the stochastic folding of chromatin and promoted liquid–liquid phase separation (LLPS) from the perspective of simultaneously boosting the transient nature and heterogeneity of nucleosome–nucleosome contacts and the effective nucleosome valency [144].

## 4. Further Mechanism Research through the Integration of Different Techniques

**Figure 3:**
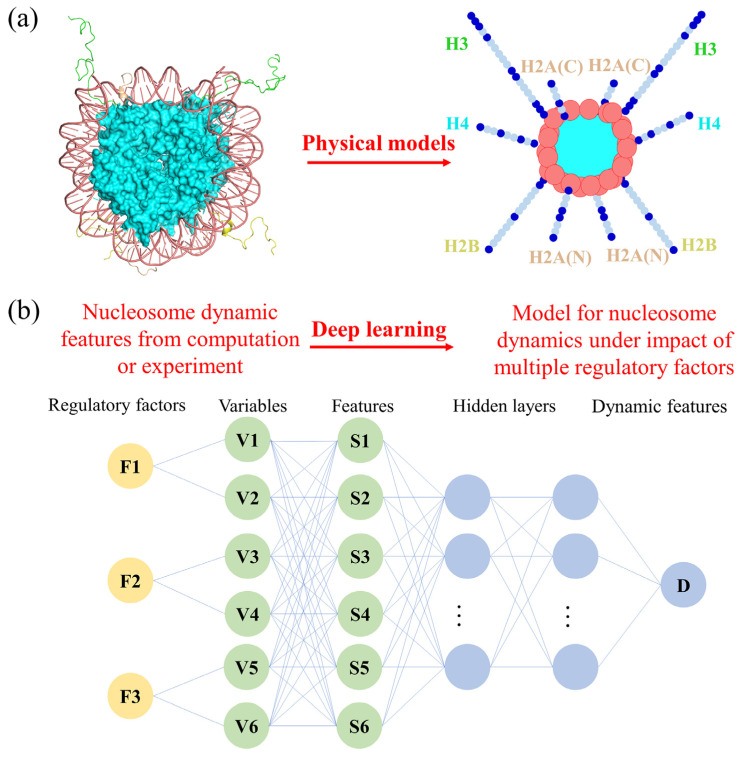
Promising approaches for the study of the mechanism of nucleosome dynamics by multiple regulatory factors. (**a**) The specific physical models of nucleosomes are depicted by the three-site-per-nucleotide and the atomic-interaction-based coarse-grained (3SPN-AICG) model. The features of nucleosome dynamics can be depicted by the physical model combined with the model force field and the equations of motion. (**b**) The deep learning method to learn the model for nucleosome dynamics under the impact of multiple regulatory factors. The multiple regulatory factors can be taken as the input features and the nucleosome dynamic features as the labeled data, and the potential relationship or comprehensive effect of different regulatory factors can be learned and applied for the prediction of a new impact.

Due to the structural and dynamic complexity of nucleosomes, any solo or combination change in the regulatory factors could have unexpected impacts on the dynamic features of the nucleosome. The current experimental or computational approaches demonstrate the general structural and dynamic modes of nucleosomes as well as the associated regulatory mechanism [145]. However, the comprehensive impact of multiple regulatory factors on nucleosomes is still needed to reflect its dynamic features under physiological conditions. Owing to the size and complexity of nucleosome structures, the temporal and spatial scales of nucleosomes or chromatin are difficult simulate at atomistic resolution with computational approaches [146]. Lequieu et al. built a new, coarse-grained model of chromatin, 1-Cylinder-per-Nucleosome (1CPN), in which the atomistic dynamics of nucleosomes were simplified by the three-site-per-nucleotide and atomic-interaction-based coarse-grained (3SPN-AICG) model (Figure 3a), the model force field, and the equations of motion controlling nucleosome dynamics. The 1CPN model incorporated physics occurring over nanometer-length scales, including histone modifications, the DNA sequence, and linker histone H1. Through the incorporation of extensive simulations with the detailed 3SPN-AICG models and experimental measurements of the structure and dynamics of chromatin, 1CPN was capable of reproducing many free energies that are involved in interactions within chromatin, including interactions between DNA and histone tails, interactions between nucleosomes, modulation by histone modifications and the salt-dependent stiffness of DNA [147]. Multiscale models such as 1CPN would be useful for studies of nucleosome dynamics. As the high degree of freedom originates from the histone variants, DNA sequence heterogeneity, and a combination of regulatory factors, a unified framework was necessary to take into account all potential impacts and construct the regulatory landscape of nucleosomes. Until now, deep learning techniques have been applied to predict the dynamic behavior of nucleosomes, and they have provided feasible strategies for the research involved in the multiple regulatory factors of nucleosomes. Gangi et al. applied a deep learning model for nucleosome identification using a sequence features representation and proved the effectiveness of the proposed method [148]. From the integrated application with molecular modeling, Ding et al. characterized the folding pathways of tetranucleosome by six inter-nucleosome distances and applied a deep learning approach to construct a six-dimensional free energy surface as a function of the inter-nucleosome distances which could support the global stability of regular fibril configurations for isolated chromatin [149]. It is supposed that by taking multiple regulatory factors as input features and nucleosome dynamic features as labeled data, the potential relationship between different regulatory factors could be learned and applied to the prediction of a new impact (Figure 3b). The construction of the model of nucleosome dynamics would require data from both experimental and computational approaches. It has been suggested that the combination of physical models and deep learning will deepen the understanding of the regulatory mechanism of nucleosomes.

## 5. Conclusions

The nucleosome is the basic unit of the chromosome’s structure, and its dynamic features in physiological circumstances determine the biological process involved. Therefore, revealing the regulatory mechanism of nucleosomes by different factors could deepen the understanding of the relationship between nucleosome dynamics and the development of diseases. In this review, a series of regulatory factors including histone modifications, DNA methylation, and nucleosome-interacting factors have been systematically analyzed and discussed. Histone modification mainly affects the interaction with the nucleosomal DNA and the interplay between different histones, while DNA methylation at CpG sites changes the physical properties of DNA and modulates the dynamics of nucleosomes. Both histone modification and DNA methylation can also change the recruitment of remodeling complexes in an indirect way. The transcription factor and ATP-dependent chromatin remodelers can bind to the surface of nucleosomes and influence the accessibility of nucleosomal DNA, and the cation concentration can change the net charge of nucleosomes and affect the assembly of nucleosomes. Although the study of the comprehensive effect of multiple factors via current computational and experimental means is quite limited due to the structural complexity of nucleosomes, further development of specific physical models for nucleosomes and the application of deep learning can provide opportunities for the further study of the nucleosome mechanism.

## Figures and Tables

**Table 1:** Overview of the current, discovered regulatory factors of nucleosome dynamics.

Regulatory Factors	Location/Subtype	Modifications ^1^/Protein Name
Histone modification	H1.2 (R54)	Me, Cit
Histone modification	H2A (Q105)	Me
Histone modification	H3 (R42)	Me
Histone modification	H3 (K56)	Me, Ac, Formyl, Succ
Histone modification	H3 (K64)	Me, Ac
Histone modification	H3 (K79)	Me, Ac, Formyl, Succ
Histone modification	H3 (T118)	Ph
Histone modification	H3 (K122)	Me, Ac, Formyl
Histone modification	H4 (K91)	Ac, Ubi, Succ, Bu, Cit, Prop
DNA Methylation	CpG	5mC
DNA Hydroxymethylation	CpG	5hmC
Transcription factor	Group I	Foxa3/Oct4/Sox2/Pu1/Ascl1/Klf4/Gata3
Transcription factor	Group IIA	Myog/cMyc/Max/Crem/Cebpα/Usf1
Transcription factor	Group IIB	Tbx1/Brachyury/NFkB p50/Gal4/TALE-PBC Pbx1/Ubx
Chromatin remodelers	ISWI	Isw1/Isw2
Chromatin remodelers	CHD	Chd1/NuRD
Chromatin remodelers	SWI/SNF	Sth1/Snf2/Swi2/Swi3/Swp73/Snf5/ARPs
Chromatin remodelers	INO80	Ino80/Swr1
Cations	monovalent	Na^+^/K^+^
Cations	multivalent	Mg^2+^/Ca^2+^/Co^3+^

^1^ The histone modifications are shown in their abbreviation: 5mC—methylation; 5hm —hydroxy methylation; Cit—citrullination; Ac—acetylation; Formyl—formylation; Succ—succinylation; Ph—phosphorylation; Ubi—ubiquitylation; Bu—butyrylation; and Prop—propionylation.

## Data Availability

Not applicable.
